# Supplementary material for: Consumers’ Perception and Preference for the Consumption of Wild Game Meat among Adults in Poland
Source: Foods. 2022 Mar 14;11(6):830. doi: 10.3390/foods11060830 (PMC8954458; doi:10.3390/foods11060830)
Supplement: Supplementary file 1 [file foods-11-00830-s001.zip › foods-1629248-supplementary.pdf]

foods MDPI

Supplementary material

Table S1. Questionnaire structure.

Question	Variants of Answers
Q1. Do you eat wild game?	Choose the right answer: (1): yes, (2): no
Q2. If you do not eat wild game, what are the reasons not to eat it? Q2.1. little availability of wild game, Q2.2. high price of wild game, Q2.3. fear of disease (e.g. trichinosis), Q2.4. no family tradition of eating game, Q2.5. lack of skill in preparing a tasty dish, Q2.6. unacceptable taste of this meat, Q2.7. I believe that good wild game dishes can only be eaten in the restaurant, Q2.8. I am not convinced that this meat is healthier than others meat, Q2.9. ethical aspects, Q2.10. other (Please specify).	Choose the right answer: (1): yes, (2): no
Q3. How often do you eat a wild game?	Choose the right answer: (1): I did not use, (2): rarely than once a year, (3): once every 2–3 months, (4): once a month, (5): two-three times a month, (6): once a week, (7): three or four times a week
Q4**.** Circumstances of wild game' dishes consumption? Q4.1. home; Q4.2. at my friends; Q4.3. catering services; Q4.4. I prepare myself; Q4.5. other (Please specify).	Choose the right answer: (1): yes, (2): no
Q5**.** What is the source of wild game you consume? Q5.1. catering establishments, Q5.2. shop / specialized wholesale, Q.5.3. typical shop / supermarket / hypermarket, Q5.4. I hunt myself, Q5.5. from the hunter (friends or family hunt), Q5.6. other (Please specify).	Choose the right answer: (1): yes, (2): no
Q6. What kind of wild game do you eat most often?	Choose the right answer: ((1): I do not use, (2): rarely than once year, (3): once a 2–3 months, (4): once a month, (5): two-three times a month, 6): once a week, (7): three or four times a week
Q7. What form do you eat wild game most often? Q7.1. wild boar, Q7.2. deer, Q7.3. roe-deer, Q7.4. hare, Q7.5. wild game birds (wild duck, pheasant, wild goose), Q7.6. other (Please specify).	Choose the right answer: (1): appetizers, (2): soups; (3): main course, (4): cold cuts and other preserves, (5): ingredients of other dishes, (6): other
Q8. If you prepare wild game dishes yourself, which method of heat treatment do you choose? 8.1. cooking, 8.2. frying, 8.3. stewing, 8.4. baking, 8.5. grilling, 8.6. raw (e.g. „wild tartare"), 8.7. smoked preserves (sausages, cold cuts and others), 8.8. non-smoked preserves (pates, brawn and others), 8.9. other (Please specify).	Choose the right answer: (0): none, (1): least often, (2): rarely, (3): moderately frequently, (4): quite often, (5) the most often
Q9. What are the reasons for your interest in consuming wild game? Q9.1. taste preferences - I like wild game, Q9.2. health properties of wild game, Q9.3. the popularity of wild game, Q9.4. meat availability, Q9.5. wild game fashion, Q9.6. family traditions, Q9.7. allergy among family members, Q9.8. hunting and the need to use the obtained meat, Q9.9. other (Please specify).	Choose the right answer: (1): yes, (2): no
Q10. Do you have concerns about the consumption of wild game?	Choose the right answer: (1): no, (2): sometimes, (3): yes

Q11. Are you hunting? If yes then how long have you been hunting?	Choose the right answer: (1): 0-5 years, (2) 6-10 years, (3): over 11 years
Gender	Choose the right answer: (1): women, (2): men
Age	Choose the right answer: (1): 18–30 years old, (2): 31–40 years old, (3): 41–50 years old, (4): >51 years old
Education	Choose the right answer: (1): vocational or primary school, (2): secondary school, (3): higher education (university)
Dwelling place	Choose the right answer: (1): village, (2): city up to 50,000 inhabitants, (3): city between 50,001–100,000 inhabitants, (4): city between 100,001–500,000 inhabitants, (5): city over 500,000 inhabitants
Financial situation in own opinion	Choose the right answer: (1): very good (2): good, (3): not good not bad, (4): bad and very bad
